# Comparison of COVID-19 and Lung Cancer *via* Reactive Oxygen Species Signaling

**DOI:** 10.3389/fonc.2021.708263

**Published:** 2021-07-02

**Authors:** Zilan Zhu, Ziyi Zheng, Jian Liu

**Affiliations:** ^1^ Department of Respiratory and Critical Care Medicine, The Second Affiliated Hospital, Zhejiang University School of Medicine, Zhejiang University, Hangzhou, China; ^2^ Zhejiang University–University of Edinburgh Institute (ZJU-UoE Institute), Zhejiang University School of Medicine, Zhejiang University, Haining, China

**Keywords:** reactive oxygen species, lung cancer, COVID-19, NRF2, HIF-1, Nf-κb

## Abstract

COVID-19 and lung cancer are two severe pulmonary diseases that cause millions of deaths globally each year. Understanding the dysregulated signaling pathways between them can benefit treating the related patients. Recent studies suggest the critical role of reactive oxygen species (ROS) in both diseases, indicating an interplay between them. Here we reviewed references showing that ROS and ROS-associated signaling pathways, specifically *via* NRF2, HIF-1, and Nf-κB pathways, may bridge mutual impact between COVID-19 and lung cancer. As expected, typical ROS-associated inflammation pathways (HIF-1 and Nf-κB) are activated in both diseases. The activation of both pathways in immune cells leads to an overloading immune response and exacerbates inflammation in COVID-19. In lung cancer, HIF-1 activation facilitates immune escape, while Nf-κB activation in T cells suppresses tumor growth. However, the altered NRF2 pathway show opposite trends between them, NRF2 pathways exert immunosuppressive effects in both diseases, as it represses the immune response in COVID-19 patients while facilitates the immune escape of tumor cells. Furthermore, we summarized the therapeutic targets (e.g., phytochemicals) on these ROS pathways. In sum, our review focus on the understanding of ROS Signaling in COVID-19 and lung cancer, showing that modulating ROS signaling pathways may alleviate the potentially mutual impacts between COVID-19 and lung cancer patients.

## Introduction

The 2019 coronavirus disease (COVID-19) is a pandemic acute respiratory disease breaking out in Wuhan and has spread throughout China and worldwide. Up to February 28th, 2021, 113,745,002 people have suffered from COVID-19 globally (1). The symptoms include fever, cough, headache, diarrhea, etc. (2). The pathogen of COVID-19 is SARS-CoV-2, an RNA virus of the *Coronaviridae* family, composed of a protein envelope and a single-stranded RNA genome (3). Spike glycoprotein (S protein) is the most distinctive protein envelope structure, crucial in the recognition and infection of the virus to host cells (3). It interacts with angiotensin-converting enzyme 2 receptor (ACE2R), expressing in multiple organs, such as lungs, heart, kidneys, intestine, brain, and testes (3). After the S protein binds to ACE2R, the virus fuses with the host cell, followed by its entry into virus genome. Replication and proliferation of the SARS-CoV-2 lead to mitochondria dysfunction, stimulating reactive oxygen species (ROS). Consequently, an aberrant cytokine storm is triggered to exacerbate inflammation, eventually causing organ damage (3).

With the highest incidence and mortality rates worldwide among various malignant tumors (4), lung cancer is a molecularly heterogeneous disease that predominantly occurs in lung epithelial cells (5). It includes two major subtypes: small-cell lung cancer (SCLC) and non-small-cell lung cancer (NSCLC), of which NSCLC accounts for about 80–85% (5). NSCLC was categorized into lung adenocarcinoma (LADC), lung squamous cell carcinoma (LSCC), and large lung cell carcinoma (6). Frequent genetic alterations have been identified in epidermal growth factor receptor (*EGFR*) and proto-oncogene *KRAS* (7, 8). Of note, targeting EGFR has been effective in treating LADC patients (9). Although LKB1 loss or IKKа mutant has been shown to drive LSCC development in mice (10, 11), no driver mutation is currently clinically validated for LSCC treatment and thus limited its targeted therapies (12). Therefore, there is an urgent need to explore new effective adjuvant drugs for patients with LSCC.

Reactive oxygen species (ROS), as critical factors involved in COVID-19 and lung cancer, is a class of vital signaling molecules produced predominantly in mitochondria by cellular metabolism to regulate several biological processes, including autophagy, immunity, and differentiation (13). An optimal level of ROS maintains oxygen homeostasis, while an imbalance between ROS production and the ability of antioxidant system to neutralize ROS leads to oxidative stress (13). Excessive ROS causes the structural and functional impairment of DNA, RNA, and protein, which is involved in disease development, such as cancer, diabetes, neurodegeneration, etc. (13). Therefore, targeting ROS and ROS-related signaling pathways is attractive.

Recently, multiple studies have shown that lung cancer patients are more prone to a deteriorated outcome and high fatality of COVID-19 (14, 15). A Meta-analysis shows that cancer patients present severer symptoms (p <0.01) and higher mortality (p = 0.03) (15) when infected with SARS-CoV-2 than those without COVID-19 infection. Besides, compared to non-cancer patients, the COVID-19 patients with cancer have significantly higher circulatory levels of proinflammatory cytokines and lower concentrations and viability of CD4 T cells and CD8 T cells (16, 17). Among the patients with all types of cancers, lung cancer patients have the second-highest risk of severe symptoms, ICU admission, and death when infected with SARS-CoV-2, indicating a possible correlation between COVID-19 and lung cancer (15). Therefore, understanding the mechanistic interplay between these two diseases will profoundly impact basic science and clinical treatment. Moreover, recent studies have revealed that ROS plays critical roles in both COVID-19 and lung cancer. Here, we compared ROS signaling pathways in COVID-19 and lung cancer, discussed how COVID-19 theoretically affects lung cancer initiation and progression *via* interacting with ROS and pointed out the promising therapeutics targeting oxidative stress for both diseases.

## ROS Systems in COVID-19 and Lung Cancer

ROS is reported to accumulate in both COVID-19 and lung cancer. This indicates the significance of understanding the homeostatic maintenance of ROS under normal conditions and the pathological alteration of ROS in COVID-19 and lung cancer. It is also essential to learn the conservative ROS alterations in both diseases to understand the interplays between them.

### ROS Systems

ROS is partially reduced O2, a highly reactive byproduct of aerobic metabolism. It comprises superoxide anion (O2^−^·), hydrogen peroxide (H_2_O_2_), and hydroxyl radical (OH∙), etc. (3). The enzymatic antioxidant systems, which primarily involve the glutathione (GSH) system and the thioredoxins (TRXs) system, keep ROS at a low level under unstressed conditions (18). Excessive ROS produced under pathological conditions disturb the balance between antioxidants and free radicals or redox, leading to a state of “oxidative stress”. The oxidative stress leads to the intracellular change of redox state and an oxidative modification of proteins (3), which is considerably associated with the pathogenesis of COVID-19 and lung cancer.

The types and levels of ROS presentation modify protein structures to regulate ROS homeostasis (18). For example, oxidative stress causes the release of TRX, an oxidoreductase activating redox-associated transcription factors, to regulate oxidative signaling pathways. The oxidized disulfide form (TRX-S2) requires the catalyst of TRX reductase (TRXR) to become functionalized (3). The reduced dithiol form [TRX-(SH2)] is active and capable of scavenging free radicals. Typically, TRX is primarily localized in the cytoplasm. In response to oxidative stress, it translocates to the nucleus to regulate the transcriptional activities by targeting Ref-1, an endonuclease promoting the DNA-binding of transcriptional factors (19).

**Figure 1:**
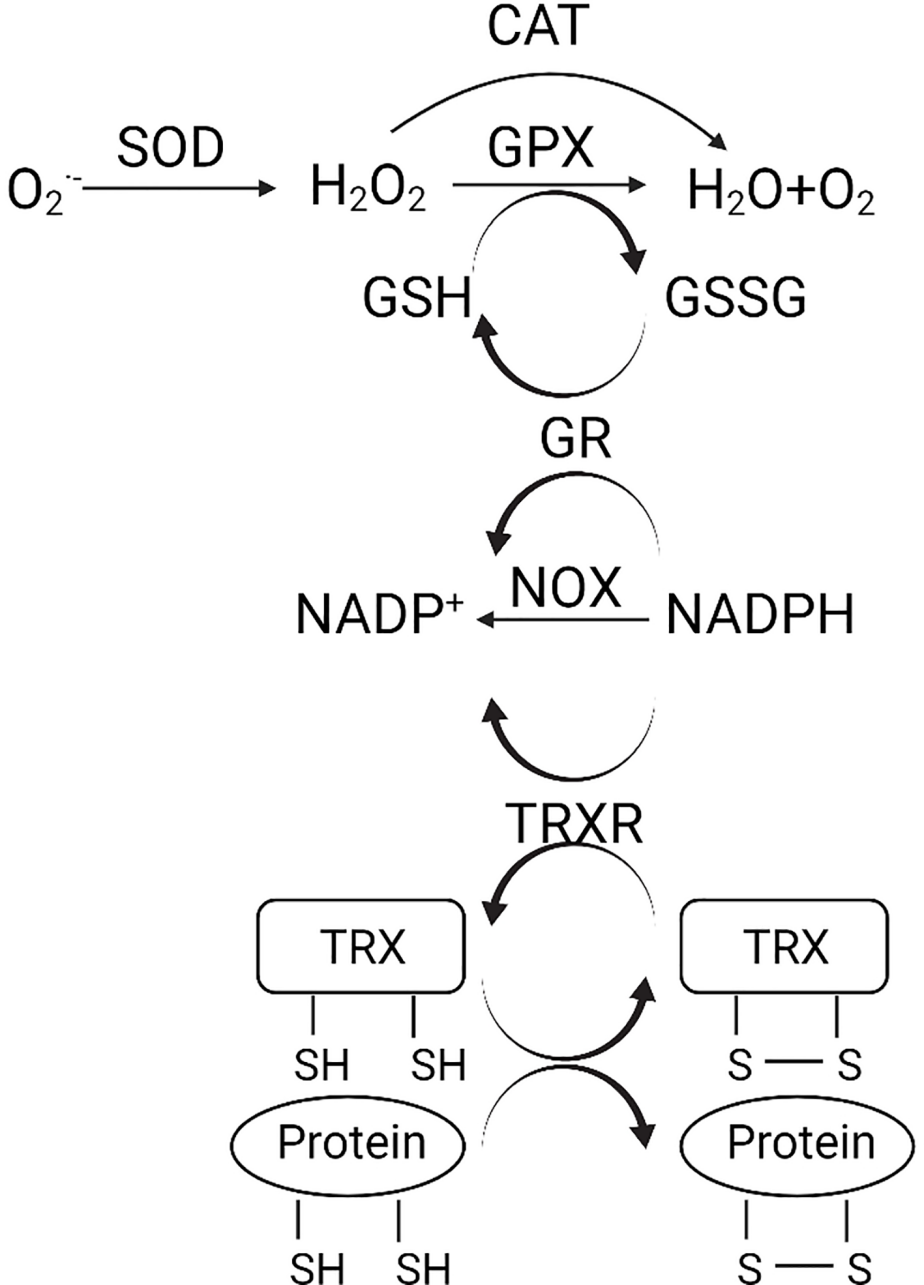
(Created with Created with BioRender.com). Schematic diagram of the antioxidant system. ${\text{O}}_{2}^{-}$, superoxide anion; H_2_O_2_, hydrogen peroxide; CAT, catalase; GPX, glutathione peroxidase; SOD, superoxide dismutase; GSH, glutathione; GSSG, oxidized glutathione; NADPH, nicotinamide adenine dinucleotide phosphate; NOX, NADPH oxidase; GR, glutathione reductase; TRX, thioredoxin; TRXR, TRX reductase.

The enzymatic antioxidant system is the predominant defense against oxidative stress (**Figure 1**). The superoxide anion radicals are decomposed to H_2_O_2_ by superoxide dismutase (SOD) (3). H2O2 is further reduced to water and oxygen catalyzed by either catalase (CAT) or glutathione peroxidase (GPX). GSH is a detoxification tripeptide that maintains the thiol status (3). Oxidative stress triggers GSH production, which reduces the superoxide, and leaves as oxidized glutathione (GSSG). Nicotinamide adenine dinucleotide phosphate (NADPH) plays a vital role in the reduction of GSSG, with glutathione reductase (GR) being the catalyst. The oxidation of NADPH is also catalyzed by NADPH oxidase (NOX) and TRXR (3).

### Dysregulated ROS Systems in COVID-19

In COVID-19, the main target of SARS-CoV-2 is ACE2R, a vital enzyme of the renin–angiotensin–aldosterone system (RAAS), which causes oxidative stress, accompanied by the viral infection, leading to the deleterious or lethal consequence (20). In the RAAS system, angiotensinII (AngII) enhances oxidative stress by stimulating NOX (20). Under normal conditions, ACE2R degrades AngII into Ang1–7, which inhibits NOX and decreases oxidative stress (21). Following the infection of SARS-CoV-2 to ACE2R, ACE2R fails to degrade AngII, consequently accumulating AngII and ROS and causing oxidative stress and cell damage (3). ROS oxidizes the S protein of SARS-CoV-2, triggering the conformational transformation of S protein and ACE2R from the reduced thiol form to the oxidized disulfide form (22). This mechanism probably increases the affinity of SARS-CoV-2 to ACE2R, which exacerbates the symptoms (3). Besides, ACE2R deficiency has been proved to increase the NOX activity in ACE2R knockout mice (23). As ACE2R in COVID-19 patients is occupied by SARS-CoV-2, failure to catalyze the AngII degradation, the NOX activity is probably increased in COVID-19 patients (3), which reduces free NADPH in circulation.

Moreover, the activation of NOX induced by COVID-19 infection may contribute to the development of pulmonary fibrosis, a typical symptom of COVID-19 infection (24). Although there is no research revealing the relationship between NADPH/NADP^+^ equilibrium and SARS-CoV-2 infection (3), the decreased NADPH concentration can impede the ROS clearance by slowing down the reduction of GSSG to GSH. The increased ROS also impairs normal endothelial function, causes excessive vasoconstriction and platelet aggregation, leading to ischemia and hypoxemia (25). Excessive oxidative stress also damages red blood cells and alveolar lung cells, which dysregulates neutrophil migration, and the local inflammation becomes global. Consequently, systemic thrombosis and atherosclerosis emerge in patients with severe COVID-19 (26).

### Dysregulated ROS Systems in Lung Cancer

Notably, the antioxidant systems and related proteins are essential for ROS regulation in cancer development. Antioxidant enzymes, such as SOD, GPX, CAT, are reduced in NSCLC compared with noncancerous lung tissues (27). GSH and its related enzymes that detoxify ROS, are accumulated in lung cancers (28). Also, the ROS-positive regulators, including NOX, are dysregulated in lung cancers. These studies indicate that targeting detoxifying reactive metabolites or antioxidant-related reactions benefits the treatment of lung cancers.

Accumulating evidence suggests that the upregulated activity and expression of the NOX family play essential pathogenic roles in oxidative stress-induced lung cancer development through ROS production (29). The NOX family member *DUOX1*, which plays a critical role in innate host defense mechanism mediated by H_2_O_2_ production and redox-dependent signaling pathways, is frequently downregulated in lung cancers (30). Moreover, one isoform, NOX4, is predominantly overexpressed and hyperactivated in lung cancer (31). Notably, NOX-derived ROS promotes lung cancer angiogenesis and tumor growth through potentiating receptor tyrosine kinase (RTK) signaling. Phosphorylated RTK activates downstream PI3K/Akt signaling upon the binding of growth factors. And then leads to the generation of superoxide anion, subsequently converted to H_2_O_2_ and other ROS (32). In turn, ROS upregulates the expression of NOX and growth factors by activating the redox-dependent transcription factors (e.g., NF-κB), forming a positive feedback mechanism (32). Inhibiting the function or expression of NOX4 *via* pharmacologic inhibitors or RNAi strategy (29) significantly blocks lung cancer progression (33). Thus, targeting tumor microenvironment by suppressing NOXs might be an effective approach for preventing and treating oxidative stress-related lung cancer.

### Conserved Changes of ROS System Components Between COVID-19 and Lung Cancer

Only a few conserved ROS system components are verified between the two diseases, probably due to a lack of understanding of COVID-19. Of note, the NOX activation is conserved in both diseases. NOX level is upregulated in both diseases, which increases oxidative stress and exerts pathogenic effects, suggesting it can be a promising target for the treatment of both COVID-19 and lung cancer (34, 35). Interestingly, GSH levels show opposite trends in COVID-19 and lung cancer. The decreased GSH level in COVID-19 infection is a consequence of decreased NADPH concentration (3). In contrast, the increased GSH level in lung cancer serves as a protective mechanism for cancer cells to survive from great oxidative stress (29).

## ROS-Related Pathways in COVID-19 and Lung Cancer

**Figure 2:**
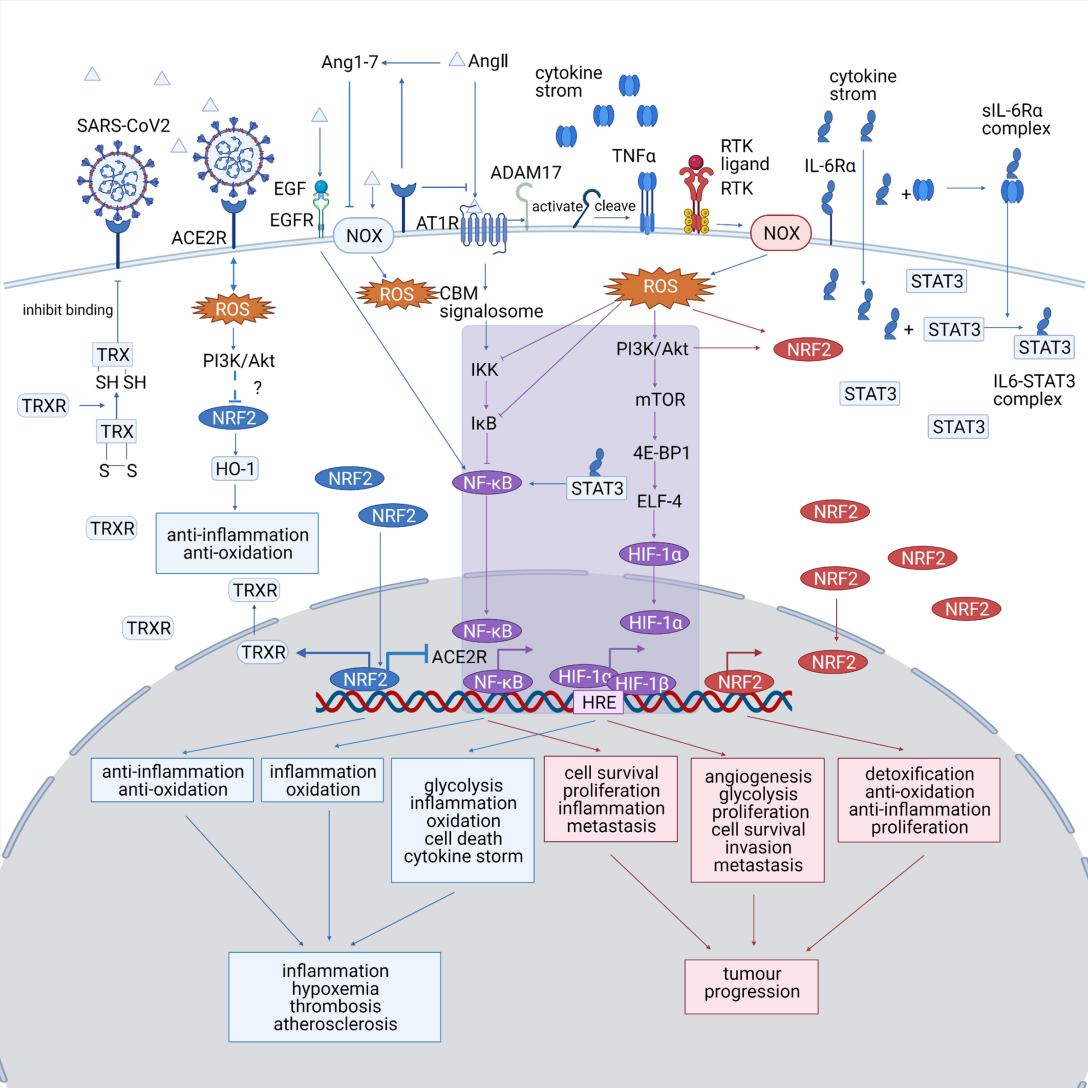
(Created with BioRender.com). A summary of the ROS-associated signaling pathways in both diseases. The COVID-19-specific pathways are marked in blue, the lung cancer-specific pathways are shown are marked in red, and the common pathways shared by both diseases are marked in purple. NRF2, HIF-1, and Nf-κB pathways play significant roles in both COVID-19 and lung cancer and probably bridges the mutual impact between them. HIF-1 and Nf-κB pathway, which are typical ROS-associated pathways, are activated in both diseases, which promote inflammation and tumor progression. The altered NRF2 pathway show opposite trends between the diseases, as it is downregulated in COVID-19, making the cells less resistant to oxidative stress, while upregulated in lung cancer, promoting the proliferation of cancer cell. NOX, NADPH oxidase; ACE2R, angiotensin-converting enzyme 2 receptor; EGF, epidermal growth factor; EGFR, epidermal growth factor receptor; Ang 1–7, angiotensin 1–7; AngII, angiotensin II; AT1R, angiotensinII type 1 receptor; TNFα, tumor necrosis factor α; IL-6, interleukin 6; STAT3, signal transducer and activator of transcription 3; ROS, reactive oxygen species; TRX, thioredoxin; HO-1, heme oxygenase-1; TRXR, TRX reductase; CBM signalosome, CARD11-BCL10-MALT1 CBM signalosome; IKK, I*κ*B-kinase; PI3K, phosphatidyl inositol 3-kinase; Akt, protein kinase B; mTOR, mammalian target of rapamycin; 4E-BP1, eIF4E-binding protein; ELF4, E74 like ETS transcription factor 4; HRE, hypoxia-responsive element.

An accumulation of ROS is detected in both diseases, which triggers cellular responses by altering redox-sensitive pathways, including NRF2, HIF-1, and Nf-κB pathways. A summary of the ROS-associated signaling pathways in both diseases is shown in **Figure 2**. ROS-relevant factors and their expression patterns, molecular functions, and potential roles in COVID-19 and lung cancer are summarized in **Table 1**.

**Table 1:** Key ROS-relevant factors and their expression patterns, molecular functions, and potential roles in COVID-19 and lung cancer.

ROS pathway	Relevant factors	Expression patterns	Molecular function	Potential roles in both diseases	Ref.
**NRF2**	PI3K/Akt	Upregulated in both diseases	PI3K phosphorylate and transfer Phosphatidyl-inositol4,5-bisphosphate (PIP2) into Phosphatidyl-inositol3,4,5-bisphosphate (PIP3), which plays critical role in Akt activation.	An upstream regulator of NRF2, probably inactivates NRF2 in COVID-19, and promotes SARS-CoV-2 entry into the host cell, while activates NRF2 in lung cancer and promotes tumor cell proliferation.	(51, 52, 125, 126)
	KEAP1	Decreased in both diseases	Negative regulator of NRF2, binds to NRF2 and facilitates its ubiquitylation.	It facilitates NRF2 upregulation in lung cancer, and promotes the tumor cell resistance to oxidative stress, while its role in COVID-19 still requires further clarification.	(37, 39, 48)
	NRF2	Activated in COVID-19, while inactivated in lung cancer	As a transcription factor, it regulates the expression of multiple antioxidant genes and viral entry sites.	In COVID-19: the inactivated NRF2 pathways downregulate HO-1 pathway, increase ACE2R expression and decrease anti-oxidase expression.In lung cancer: promotes aggressive proliferation, metastasis of tumors, and tumor resistance to oxidative stress, chemo- and radiotherapy.	(38, 39)
	HO-1	Inactivated in COVID-19	HO-1 degrades heme into biliverdin, iron, and carbon monoxide. Biliverdin is then converted into bilirubin, which has anti-inflammatory, anti-apoptotic, anti-thrombotic, anti-fibrotic, and anti-edema effects.	Increases oxidative stress and magnifies the harmful effect of ROS.	(39, 40)
**HIF-1 and hypoxia**	mTOR	Activated in both diseases	Activate 4E-BP1	Promote viral replication, angiogenesis, tumor cell proliferation, inhibit apoptosis	(127, 128)
	4E-BP1/ELF-4	Over-expressed in both diseases	4E-BP1 is an mTOR-sensitive protein, which binds to ELF-4 to inhibit the translation initiation of HIF-1α.	Promote tumor cell proliferation and repress protein expression	(58, 129)
	HIF-1	Over-activated in both diseases.	HIF-1 is a transcriptional regulator, controlling the expression of glycolytic genes and facilitates glycolysis	Promote ROS production and increase oxidative stress. Trigger cytokine storm and excessive immune response. Regulate key adaptive mechanisms including glycolysis and angiogenesis, and that drive pro-survival signaling, cell proliferation and metastasis in cancers	(57, 66, 72, 130)
**NF-kB**	metalloprotease 17 (ADAM17)	Activated in COVID-19	Mediate the splicing of TNFα and sIL-6Rα	Triggers cytokine storm.	(75)
	sIL-6Rα	Accumulated in COVID-19	A combination of TNFα and IL-6Rα	Transduces signal.	(75)
	CBM signalosome	Activated in COVID-19	A combination of CARD and membrane-associated guanylate kinase-like protein, B-cell lymphoma 10, and mucosa-associated lymphoid tissue lymphoma translocation protein 1. It is activated by the binding of AngII to AT1R, and activates IκB kinase complex.	Transduces signal.	(76)
	IL-6	Accumulated in COVID-19	Binds to and activates STAT3.	Triggers cytokine storm and inflammation. Amplifies NF-κB signaling.	(75)
	STAT3	Activated in COVID-19	Promote IL-6 transcription.	Triggers cytokine storm and inflammation. Amplifies NF-κB signaling.	(75)
	IKK	Repressed in both diseases	Phosphorylate, ubiquitylate, and degrade IκB.	Transduces signal.	(76, 91)
	IκB	Repressed in both diseases	Inhibit NF-κB activation.	Transduces signal.	(76, 91)
	NF-κB	Over-activated in both diseases	Regulate downstream antioxidant and pro-oxidant targets to affect intracellular ROS amounts.	In COVID-19: increases oxidative stress, triggers cytokine storm, promotes inflammation.In lung cancer: promotes tumor cell proliferation, metastasis, and inflammation.	(77, 90, 92)

### Transcription Factor NRF2

The nuclear factor erythroid-2-related factor 2 (NRF2) is a vital redox-sensitive transcription factor, controlling cellular antioxidant responses *via* regulating the expression of GSH metabolism-related enzymes and enzymatic antioxidant systems and their cofactors (NADPH, FADH2) (36). NRF2 expression levels are usually kept low during unstressed conditions in all cell types; while in response to oxidative stress, NRF2 activation results in transcriptional upregulation of a wide range of enzymes involved in xenobiotic detoxification, antioxidant response, and maintenance of cellular redox homeostasis (36). Kelch-like ECH-associated protein 1 (KEAP1) is an adapter protein of the CUL3 ubiquitin ligase that negatively regulates the protein level of the critical stress response mediator NRF2 (37). Cellular ROS level is usually regulated by NRF2 and its repressor KEAP1, which promotes NRF2 degradation by the proteasome (37). In response to oxidative stresses, conformational change of KEAP1 leads to nuclear translocation of accumulated NRF2 (37). NRF2 activates the transcription of genes involved in defenses against ROS (38).

#### NRF2 in COVID-19

In COVID-19 patients, Heme Oxygenase 1 (HO-1) pathway induced by NRF2 was repressed in the Vero-hTMPRSS2 cells infected by SARS-CoV-2 (39). HO-1 protects cells from inflammation and oxidative stress (40). Moreover, NRF2 activation downregulates ACE2R expression in respiratory epithelial cells (41), minimizing the entry sites of SARS-CoV-2. Additionally, NRF2 activation promotes TRXR expression, which activates TRX and decreases oxidative stress (42). TRX reduces the disulfide bonds of SARS-CoV-2 and ACE2R, potentially impairing the binding of the ligand to the receptor and thus inhibiting COVID-19 progression (43).

Besides, the repressed NRF2 pathways also deteriorate inflammation by upregulating proinflammatory cytokines and chemokines and recruiting immune cells. As NRF2 represses the transcriptional expression of IL-1β, IL-6, and TNFα *via* inhibiting the recruitment of RNA polymerase II in macrophages under unstressed conditions (44), its downregulation promotes the proinflammatory transcription (45). Moreover, the repressed NRF2 pathways promote the production of proinflammatory cytokines *via* activating the macrophage NLRP3 and AIM2 inflammasome to recognize pathogen-associated molecular patterns (PAMPs) (46, 47). Thus, activating NRF2 and related pathways (e.g., HO-1) may attenuate the effect of SARS-CoV-2 infection and reduce the inflammatory response.

#### NRF2 in Lung Cancer

Large-scale genomic studies have revealed alterations of the KEAP1*/*NRF2 pathway in 23% of LADC and 34% of LSCC, approximately (48). Constitutive NRF2 activation and subsequent ROS suppression resulted from *Keap1* deletion in a mouse model of LSCC promote aggressive proliferation, metastasis of tumors, and tumor resistance to oxidative stress, chemo- and radiotherapy (38). Under oxidizing conditions, high intracellular ROS promotes the dissociation of NRF2 and KEAP1 *via* activating PI3K (49). Then PI3K phosphorylates NRF2 to upregulate the expression of metabolic genes promoting cancer cell proliferation (49). Moreover, sustained PI3K signaling with NRF2 pathway activation may promote lung tumorigenesis (50). These imply that targeting PI3K and NRF2 is promising to treat lung cancer.

One study showed that combined loss of *Pten* and *Keap1* promotes the formation of LADC in mice. Sustained NRF2 activation induced by loss of *Pten* and *Keap1* leads to reprogrammed cellular pentose phosphate pathway (PPP) and an immunosuppressive microenvironment, characterized by specific upregulation of programmed death ligand-1 (PD-L1) on tumor cells and an enhanced expression of PD-1 on CD8 T cells (50). As an important immune checkpoint, the PD-1/PD-L1 pathway can be exploited in lung cancer therapeutics. Notably, combinational immunotherapy of anti-PD-1/anti-CTLA-4 treatment resulted in tumor regression, associated with the increased numbers of infiltrating lymphoid cells, robust T cell activation, and reduced hyperplasia in tumor-bearing lungs (50).

#### NRF2 in COVID-19 and Lung Cancer

The activity of the KEAP1/NRF2 pathway is distinct in COVID-19 and lung cancer. In COVID-19, NRF2 is inactivated and its associated genes are downregulated. Although no evidence has shown relevance between PI3K/Akt pathway and NRF2 in COVID-19, PI3K/Akt is probably associated with the inactivated NRF2, as such mechanism has been verified in chronic obstructive pulmonary disease (51). The suppressed NRF2 pathway represses its downstream HO-1 pathway and decreases the protective effects against ROS, leading to overloaded oxidative stress and deteriorated inflammation (39). Contrarily, NRF2 is overexpressed as a result of excessive KEAP1 deletion and PI3K/Akt activation in lung cancer, which results in the resistance to oxidative stress and causes uncontrollable proliferation and metastasis of tumor cells (52). The difference of NRF2 expression in the two diseases might be associated with PI3K/Akt pathway, as it possibly inactivates NRF2 in COVID-19 while activates NRF2 in lung cancer. Thus, mechanisms of how PI3K/Akt regulates NRF2 expression require further clarification. NRF2 pathways exert immunosuppressive effects in both diseases, as it represses the immune response to SARS-CoV-2 in COVID-19 patients and facilitates the immune escape of tumor cells (45, 50).

### HIF-1 and Hypoxia

Hypoxia is a common characteristic shared in both COVID-19 and lung cancer due to altered energy metabolism caused by constant oxidative stress and diminished oxygen and nutrient availability (53). The cells tend to adapt anaerobic metabolism, known as aerobic glycolysis or the Warburg effect, to survive in hypoxia (54). A central hypoxic signaling pathway is the activation of hypoxia-inducible factor-1 (HIF-1) that is composed of HIF-1α and HIF-1β subunits (55). Under hypoxic conditions, HIF-1α dimerizes with HIF-1β and binds to hypoxia response element (HRE) to regulate glycolysis and angiogenesis, drive pro-survival signaling and cell proliferation (55).

#### HIF-1 and Hypoxia in COVID-19

In COVID-19 patients, hypoxia and ROS both promote the transcription and stabilization of HIF-1α (56, 57). Detailed mechanism of HIF-1α upregulation is still unclear, but it is hypothesized to be regulated by the PI3K/Akt/mTOR pathway. In detail, PI3K activation leads to Akt phosphorylation, which consequently activates mTOR. Downstream signaling cascades are then triggered, including activation of the 4E-BP1 and ELF4 complex and HIF-1α expression (58). Consequently, HIF-1α promotes the expression of glycolytic genes and facilitates glycolysis, which generates ATP and triggers the release of calcium ions, thereby facilitates ROS synthesis (57). Glycolysis is also necessary for the replication and proliferation of SARS-CoV-2 and triggers monocyte inflammatory response (56). The metabolic change of proinflammatory monocytes upregulates the expression of cytokines, such as TNF-α, IL-1β, and IL-6, leading to the cytokine storm. Consequently, it leads to T cell dysfunction and lung epithelial cell death (56). The PI3K/Akt/mTOR/HIF-1α pathway has been verified in various diseases associated with inflammation, such as allergic airway inflammation (59). However, the only research focus on the PI3K/Akt/mTOR/HIF-1α in COVID-19 pathogenesis (60) is in contradiction to the evidence of overexpressed HIF-1α in other studies (56, 57). The Akt-mTOR expression was upregulated, while downstream expression of HIF-1α was suppressed in human hepatocyte-derived cellular carcinoma cell line Huh7 infected by SARS-CoV-2 (60). Thus, further study is still required to clarify the role of PI3K/Akt/mTOR pathway in regulating HIF-1α expression in COVID-19.

Besides, HIF-1α plays a key role in regulating the immune and inflammatory response. It activates dendritic cell and neutrophil activation by increasing glycolysis (61, 62). Moreover, it stabilizes the M1 signal required for macrophage stabilization and polarization by regulating the expression of glucose transporters (63, 64). HIF-1α also delays the exhaustion of neutrophils by downregulating the transcription of proapoptotic mediators, such as Sival (65, 66). However, overactivation of HIF-1α leads to tissue damage and organ failure, including acute lung injury, due to the exacerbation of cytokine storm and inflammation (66).

#### HIF-1 and Hypoxia in Lung Cancer

Regulation of HIF-1α is a crucial way of hypoxia-induced metabolic reprogramming. Although prolonged lack of oxygen inhibits normal cell metabolism, hypoxia promotes glycolytic phenotype in tumor cells *via* stabilizing HIF-1α (67). HIF-1α is usually overexpressed in NSCLC (68). In NSCLC, hypoxia-induced activation of PI3K/Akt/mTOR signaling further activates the 4E-BP1 and ELF4 complex and promotes HIF-1α expression (69). The accumulated HIF-1α increases ROS levels and converts cellular metabolism into aerobic glycolysis under prolonged hypoxia, promoting metastasis of lung cancer cells (53). In NSCLC, cancer cells maintain their vitality under hypoxic conditions by restraining ROS production in the mitochondrial oxidation respiratory chain (70). The Warburg effect induced by HIF-1α activation promotes cell proliferation and tumor growth (71). Besides, hypoxia-induced HIF-1 increases the expression of PD-L1 on the cell surface. Overexpressed PD-L1 enables tumor cells to escape from immune system surveillance *via* binding to PD-1 expressed by T cells, thus preventing tumor-infiltrating T cell activation and promoting tumor cell survival (67).

#### HIF-1 and Hypoxia in COVID-19 and Lung Cancer

COVID-19 infection might contribute to lung cancer development *via* HIF-1-associated pathways. SARS-CoV-2 activates the PI3K/Akt/mTOR signaling pathway, which probably increases HIF-1α expression and promotes cancer development (59). On the other hand, the hypoxic and inflammatory microenvironment of COVID-19 can also induce hypoxemia in patients and directly upregulate HIF-1α expression. As the overexpressed HIF-1α can result in the occurrence, angiogenesis, invasion, and metastasis of lung cancer (72), we hypothesized that COVID-19 infection might promote lung cancer development *via* HIF-1α-associated pathways. Moreover, the hypoxia-dependent immune system escape based on the PD-1/PD-L1 pathway in lung cancer might also occur in COVID-19 (67). Thus, further studies on hypoxia and HIF-1 can gain insight into COVID-19 and lung cancer treatment.

### Inflammation and NF-κB

The metabolic dysregulation and hypoxic microenvironment due to oxygen and nutrient depletion in tumor propagation often lead to inflammation, facilitating COVID-19 and lung cancer progression (73). Nuclear factor-kappa B (NF-κB) is a family of crucial transcription factors, acting as vital mediators in inflammatory responses and tumor-progression mechanisms *via* multiple pathways. NF-κB regulates downstream antioxidant and pro-oxidant targets to affect intracellular ROS amounts. Recent evidence has indicated that the expression of TRX1 and TRX2, the two most critical cellular antioxidants, can be upregulated by NF-κB, thus protecting cells from ROS-induced oxidative stress (74).

#### Inflammation and NF-κB in COVID-19

The oxidative environment in COVID-19 can activate the redox-sensitive NF-κB *via* AngII-AngII Type 1 Receptor (AT1R) axis (75). On the one hand, the AngII-AT1R axis activates metalloprotease 17 (ADAM17) to induce the activation of NF-κB upstream regulators, such as epidermal growth factor and TNFα (75). Additionally, ADAM17 also mediates the activation of Signal Transducer and Activator of Transcription 3 (STAT3) to induce NF-κB activation in IL-6Rα-negative nonimmune cells (75). ADAM17 can modify IL-6Rα to form a sIL-6Rα-IL-6 complex which promotes the formation of IL6-STAT3 complex, finally activating its downstream NF-κB (75). In turn, the activated STAT3 and NF-κB further stimulate the IL-6 amplifier to activate NF-κB, thus forming a positive feedback loop that leads to deteriorated inflammation (75). On the other hand, the AngII-AT1R axis promotes the formation of membrane-associated guanylate kinase-like protein, B-cell lymphoma 10, and mucosa-associated lymphoid tissue lymphoma translocation protein 1 (CBM) signalosome (76). CBM signalosome activates IκB kinase complex to induce the phosphorylation and degradation of IκB, leading to NF-κB activation (76).

Secondly, NF-κB induces the expression of pro-inflammatory cytokines, stimulated by pro-oxidant cytokines produced in the AngII-AT1R axis, such as TNF (77). The cytokine storm triggers ROS production primarily through the respiratory burst activity of macrophages, monocytes, and neutrophils (77, 78). Moreover, the cytokine storm driven by NF-κB pathways can trigger neutrophil extracellular traps (NET) formation (79, 80), suggesting the programmed death of neutrophils (81). NET contains ample cationic enzymes that lead to cell lysis, tissue damage, and local or systemic inflammation (82, 83).

NF-κB is suggested to be a vital determinant of the severity of COVID-19 (84). Aging-related upregulation of NF-κB expression, as a redox-sensitive transcription factor associated with the proinflammatory condition, causes an extreme immune response in aged non-human primates infected with SARS-CoV (85). A recent study shows that the old are more vulnerable to SARS-CoV-2 infection and present more severe symptoms (86). This is because of antioxidant system deprivation, increased ROS level, and ROS susceptibility (87, 88). Since the infection effects of the SARS-CoV and SARS-CoV-2 on ROS and its associated pathways are conserved, ROS level affects the severity of COVID-19 (89). It may serve as a diagnostic biomarker to distinguish the infection stage of COVID-19.

#### Inflammation and NF-κB in Lung Cancer

Depending on the cellular context, ROS plays either inhibitory or stimulatory roles in modulating upstream or downstream targets of NF-κB transcriptional activity (74). For instance, ROS could disturb NF-*κ*B activity *via* inhibiting I*κ*B activation (90). Secondly, IKK*β*, the upstream kinase phosphorylating and activating I*κ*B, is another major target of ROS, whose activity is inhibited as ROS induces the S-glutathionylation of IKK*β* on cysteine179 (90). Furthermore, canonical NF-*κ*B-activating pathway relies on two processes, the phosphorylation and activation of IKK*β*, as well as the ubiquitination and degradation of phosphorylated IκB, both of which have interrelation with ROS (74).

NF-κB pathway plays a crucial role in regulating inflammation and mediating immune surveillance in lung cancer, especially promoting antitumor T cell responses. Enhanced NF-κB activity leads to tumor rejection and suppresses tumor growth *via* upregulating the expression of several T cell chemokines, including CCL2, CCL5, and recruiting cytotoxic CD8 T cells (91).

#### Inflammation and NF-κB in COVID-19 and Lung Cancer

Emerging evidence suggests that COVID-19 may affect a particular stage in the life cycle of tumor cells *via* NF-κB pathway, especially the dormant cancer cells (DCCs). DDCs often localize in a quiescent state as metastatic dormancy (92) and can be reactivated by microenvironmental cues, including inflammatory and immune-mediated signals in COVID-19 infection, to initiate and progress metastasis.

During the severe COVID-19 infection, elevated IL-6 and other released pro-inflammatory cytokines lead to a widespread activation of NF-κB in both immune and non-immune cells (93). Activated NF-κB in lung inflammation would then trigger DCCs reawakening *via* directly stimulating cancer cell proliferation and indirectly inducing the formation of a pro-metastatic microenvironment (93). Additionally, clinical studies on long-term effects of COVID-19 in cancer patients will clarify the relationship between COVID-19 infection and the risk of pulmonary metastatic recurrence (94). If the correlation is confirmed, anti-inflammatory agents interfering with the immune-mediated NF-κB pathways might be helpful in the prevention of subsequent tumor relapse. Thus, NF-κB and components of NF-κB-related pathways might be potent and important targets in therapeutics for both COVID-19 and lung cancer.

## Drug Targets in ROS-Related Pathways

Besides traditional treatments for the two diseases (i.e., immunotherapy for COVID-19 and chemotherapy and radiotherapy for lung cancer), modulation of intracellular oxidative stress has emerged as a potential treatment. ROS-related pathways (e.g., NRF2, HIF-1, and NF-κB, etc.) might become therapeutic targets. Hence, current understanding of potential therapies related to these pathways will be mainly discussed below. ROS-modulating compounds and their effects in reversing COVID-19 and lung cancer are summarized in **Table 2**.

**Table 2:** ROS-modulating compounds and their effects in reversing COVID-19 and lung cancer.

Category	ROS-modulating treatments	Suggested target disease	Effect in treating COVID-19	Ref.
**NRF2 mediator**	Curcumin	COVID-19 & Lung cancer	Decreases immune cell infiltration, suppresses proinflammatory responses, decreases oxidative stress, inhibits tumor progression	(95, 98, 99)
	Bis [2-hydroxybenzylidene]acetone (BHBA) (an curcumin-derivative)	Lung cancer	Decreases oxidative stress, represses inflammation and tumor progression	(98)
	Resveratrol (RSV)	COVID-19 & Lung cancer	In COVID-19: Activates NRF2 pathways and decreases ROS levels and cell apoptosisIn lung cancer: inactivates NRF2 pathways, inhibits tumor proliferation and metastasis	(104, 105)
**HIF-1α inhibitor**	Curcumin	COVID-19	Decreases immune cell infiltration, suppresses proinflammatory responses, decreases oxidative stress	(95, 98, 99)
	miR-130a	COVID-19 & Lung cancer	In COVID-19: probably regulates glucose and energy metabolism, alleviating the negative effect of ischemia and hypoxiaIn lung cancer: suppresses the Warburg effect, NSCLC cell metastasis	(110, 112)
	miR-200c	Lung cancer	Inhibits lung carcinoma cell metastasis	(111)
	miR-199a	Lung cancer	Suppresses NSCLC cell proliferation	(68)
	RSV	COVID-19	Inhibits HIF-1α translation	(105)
	MK-2206	COVID-19	Akt inhibitor	(60)
	rapamycin	COVID-19	mTORC1 inhibitor	(60)
	Torin-1	COVID-19	mTORC1&2 inhibitor	(60)
	PX-478	COVID-19	HIF-1α inhibitor	(60)
	Chloramphenicol	Lung cancer	Induces mitophagy, destabilizes HIF-1α	(113)
	alteration of oxygen exposure	COVID-19 & Lung cancer	In COVID-19: intermittent hypoxia/normoxia or hypoxia/hyperoxia training, which promotes mitochondria biogenesis, prevents apoptosis, reduces oxidative stressIn lung cancer: hyperoxia or hypoxia treatment, which increases ROS activity, promotes tumor cell apoptosis, increases blood oxygen	(55, 106, 107, 109)
**NF-κB inhibitor**	Bortezomib	Lung cancer	Prevents IκB protein degradation, inhibits tumor progression	(114)
	Vitamin D	COVID-19	Facilitates IκB expression, causes the death of infected cells	(97)
	Calcitriol (a Vitamin D analog)	COVID-19	Facilitates IκB expression, causes the death of infected cells, reduces ROS level	(115)
	miR-21 inhibitor	COVID-19 & Lung cancer	In COVID-19: decreases the expression of ACE2In lung cancer: suppresses tumor migration and invasion and promotes cell apoptosis	(118, 119)
	NF-KappaB Interacting LncRNA (NKILA)	Lung cancer	inhibits IκB phosphorylation and NF-κB activation, suppresses tumor metastasis in NSCLC	(121)
	Flavonoid	COVID-19 & Lung cancer	In COVID-19: blocks the binding site of SARS-CoV-2Induces apoptosis, inhibits proliferation and metastasis, reduces ROS level	(122–124)

### Drug Targets in NRF2-Related Pathways

#### Regulation of NRF2 Through the KEAP1-NRF2 Feedback Loop

Though the NRF2 pathway seems to play opposite roles in COVID-19 and lung cancer, it is still a viable target for lung cancer patients with COVID-19. Curcumin, an active polyphenolic compound of the *Curcuma longa* plant, is a promising antioxidant, anti-inflammatory, and anticancer agent (95) that probably benefits the therapy of both diseases. It activates the NRF2/KEAP1 pathway while represses NF-κB-mediated pathways (95). Curcumin may benefit the treatment for COVID-19, as it decreases the infiltration of immune cells and suppresses the proinflammatory responses (96). Besides, it can either directly clear ROS or indirectly reduce oxidative stress *via* increasing SOD, which transforms superoxide anion into H_2_O_2_, and then reduced it in GSH antioxidant system (97). Curcumin induces cancer cell apoptosis and inhibits growth, proliferation, and invasion of tumors *via* ameliorating various cellular responses to oxidative stress (98). One curcumin-derivative, bis [2-hydroxybenzylidene]acetone (BHBA), broadly protects human lung epithelial cells against cytotoxicity *via* potently inducing NRF2 activation in KEAP1-dependent manner (98). As clinical trials have already shown remarkable protective and therapeutic effects of curcumin in oxidative-associated liver disorders (99), its usage in lung cancer deserves future study. Of note, activation of the NRF2 expression in COVID-19 patients with lung cancer using the non-selective drug delivery system should be under a delicate tone manner as the overactivated NRF2 pathway is carcinogenic (100). Moreover, nanomaterial delivery systems can be utilized to further finely regulate NRF2 expression by specifically inactivating NRF2 pathways in cancer cells (101). In detail, those nanomaterials with surface modification become highly bio-sensitive and can get through the “biological barriers”, such as the tumor cell membrane penetration and the attack of the immune system (102, 103). And then, the modified nanomaterials recognize biomarkers identifying the cell type and then release cargoes in an intelligence-controllable manner (102, 103). Therefore, although the NRF2 pathway appears to play opposite roles in COVID-19 and lung cancer, targeting the NRF2 pathway by applying nanomaterial delivery systems can be a viable approach for lung cancer patients with COVID-19 symptoms.

#### Regulation of NRF2 *via* PI3K/Akt Signaling Pathway

Resveratrol (RSV), a plant-derived polyphenol acting as a vital antioxidant, can protect various organs from oxidative stress at least partially *via* the PI3K/Akt-mediated NRF2 signaling pathway (104). The potential role of RSV in preventing and treating COVID-19 has been suggested by various studies (105). Studies revealed that RSV pretreatment directly increases cell viability and expression of SOD, CAT, and GPX, while decreases intracellular ROS levels and cell apoptosis. Moreover, RSV significantly attenuates H_2_O_2_-induced intestinal cell damage from oxidative stress through upregulating phosphorylated Akt levels to activate NRF2 (104). Collectively, these findings have shown cytoprotective effects of RSV against oxidative stress, which may also be a potentially effective agent in lung cancer therapy.

### Treatments Related to Hypoxia and HIF-1 Pathways

#### Through the Alteration of Oxygen Exposure

The optimal hypoxic pre-conditioning training is possibly beneficial for of COVID-19 patients (57). After intermittent hypoxia/normoxia or hypoxia/hyperoxia training, the niche becomes adaptive to hypoxic state and HIF-1α expression is promoted (57). Consequently, the expression of downstream protective factors, such as antioxidant enzymes, is increased (106). Besides, HIF-1α reverses acute respiratory diseases by promoting angiogenesis, mitochondrial biogenesis, and preventing apoptosis (55).

For lung cancer, recent studies have focused on reversing the hypoxic state in tumors. Hyperbaric oxygen (HBO) treatment increases blood oxygen and retardates tumor growth (107). However, oxygen toxicity may be harmful to the central nervous system (108). An alternative of hyperoxic treatment is normobaric hyperoxia (NBO), a feasible therapy that increases ROS activity and cancer cell apoptosis with a low complication rate, easy administration, and non-invasiveness (109).

#### Through the Gene Expression Products That Inhibit HIF-1α

Long noncoding RNAs (lncRNAs) regulates gene expression, which probably influences the COVID-19 progression by regulating HIF-1α (110). HOTAIR decoys miR-130a-3p and alters HIF-1α in hepatocellular carcinoma (110). Although the role of lncRNA in SARS-CoV-2 infection is still unclear, it at least brings us new thoughts to studying the mechanism of COVID-19.

miRNAs precisely regulate NSCLC progression and metastasis. Studies have reported that several miRNAs prevent the hypoxia-induced proliferation of NSCLC cells through targeting HIF-1α. In response to hypoxia, miR-200c negatively regulates hypoxia-induced cellular responses by downregulating HIF-1α, leading to a decreased mRNA level of HIF-1α downstream genes and an inhibited metastasis of lung carcinoma cells (111). Additionally, ectopic expression of miR-130a suppresses the Warburg effect, migration, and invasion of NSCLC cells under hypoxia *via* targeting HIF-1α (112). NSCLC cells with low miR-199a levels have higher HIF-1α expression and proliferation capacity, while overexpressed miR-199a suppresses the hypoxia-induced cell proliferation through silencing HIF-1α expression and blocking HIF-1α-mediated glycolytic pathways (68). Therefore, beneficial regulation of miRNAs on HIF-1α strengthens their tumor-suppressive activity, suggesting that miRNAs or their mimics may serve as anticancer agents through inhibition of tumor metastasis and multiple hypoxia-induced responses.

#### Through Plant Metabolites and Their Synthetic Derivatives Targeting HIF-1-Related Pathways

Inhibitors to HIF-1α and HIF-1-associated signaling pathways are promising in reversing both diseases. RSV inhibits hypoxia-mediated overexpression of HIF-1α, thus might have a protective effect on COVID-19 (105). Inhibitors to the Akt/mTOR/HIF-1 signaling are also promising in treating COVID-19. MK-2206 (Akt inhibitor), rapamycin (mTORC1 inhibitor), Torin-1 (mTORC1&2 inhibitor), and PX-478 (HIF-1α inhibitor), significantly downregulates viral transcripts in SARS-CoV-2 infected Huh7 cell culture, indicating they could be repurposed and potentially used to treat COVID-19 (60).

Although potent inhibitors directly targeting HIF-1α pathways against hypoxic tumors are still limited, chloramphenicol, an inexpensive and excellent bactericidal antibiotic, has been found to inhibit HIF-1α accumulation in a concentration-dependent manner in NSCLC (113). Chloramphenicol not only induces autophagy but also prevents the formation of HIF-1α/SENP-1 (Sentrin/SUMO-specific-protease-1) protein complex, essential for HIF-1α stabilization during hypoxia (113). Both inhibitors destabilize HIF-1α protein and promote their degradation (113).

### Therapeutic Potential of Targeting NF-*κ*B

#### NF-κB Inhibitors Targeting Kinase Activity

In recent years, advancements have been made in developing and characterizing both natural and synthetic agents blocking NF-κB activity. Those NF-κB inhibitors exert anti-tumor or antiviral effects in lung cancer or COVID-19 mainly affecting NF-κB induction, NF-κB nuclear translocation, and DNA binding (90), all of which have interrelation with ROS as indicated above.

Various IKK inhibitors have been developed to inhibit the kinase activity of IKK directly or block upstream proteins targeting IKK to prevent IKK activation indirectly. Proteasome inhibitors are used to interfere with IκB protein degradation, thus blocking NF-κB activation. For example, Bortezomib, a proteasome inhibitor, has shown beneficial antitumor outcomes with manageable side effects. Moreover, clinical trials have demonstrated high anticancer efficacy and better responses when combining Bortezomib with other anticancer drugs in NSCLC, such as EGFR/HER2-targeting agent cetuximab (114).

Vitamin D and its analogs have therapeutic effects against COVID-19, as its binding with vitamin D receptor facilitates IκB expression and blocks NF-κB activation (97). Consequently, uncoupling protein-2, a downstream target of NF-κB, is downregulated, which leads to cell death (97). Calcitriol, an active form of vitamin D, was also found to reduce ROS *via* increasing the concentration of GSH, SOD, and CAT (115). However, one caveat is that blocking or deregulation of NF-κB signaling may compromise immunity since systemic administration of NF-κB inhibitors may deteriorate the protective immune responses (116).

#### Combinational Therapy Using Chemotherapeutics With NF-κB Inhibitors

Currently, a study shows that using chemotherapeutics in combination with NF-κB inhibitors seems to be a preferred approach for cancer treatment, especially for tumors with chronic inflammation (73). Indeed, combinational therapy using both NF-κB inhibitors and inhibitors of other transcription factors, such as STAT-3, has shown effective antitumor outcomes, as the STAT-3 signaling pathway that fuels tumor promotion and mediates immune escape has important crosstalk with NF-κB (117). Since STAT-3/NF-κB signaling plays an important role in deteriorated inflammation in SARS-CoV-2 infection, the combination therapy may also show potential outcomes in COVID-19 treatment. Additionally, further understanding of upstream regulators and downstream effectors within NF-κB pathways might identify more selective targets for antiviral therapy and preserve effective antitumor immunity (117).

#### NF-κB Inhibitors Targeting NF-κB-Related Pathways *via* miRNA Regulation

Increasing evidence has demonstrated that many miRNAs play important roles in COVID-19 and lung cancer progression *via* NF-κB-related pathways (118, 119), serving as potential diagnostic markers, prognostic markers, and therapeutic targets. Upregulation of miRNAs, such as miR-21, is associated with the initiation and development of NSCLC (118). Meanwhile, miRNAs regulate the expression of the viral genome and even hijack the host gene expression during SARS-CoV-2 infection (119). For instance, miRNA is largely associated with the expression of ACE2R, the entry site of SARS-CoV-2 (119). Furthermore, miR-21 downregulation induced by miR-21 inhibitor was found to suppress tumor migration and invasion and promote cell apoptosis in NSCLC through inhibiting PI3K/Akt/NF-κB signaling (120). Moreover, *NF-KappaB Interacting LncRNA* (NKILA) suppresses tumor metastasis in NSCLC *via* NF-κB/Snail pathway, in which activated NKILA inhibits IκB phosphorylation and NF-κB activation (121). Indeed, NF-kB inhibitors are used to reverse NKILA-regulated malignancy (121). These findings collectively reveal novel mechanisms of miRNA and noncoding-RNAs in NF-κB-related pathways and provide potential targets in NSCLC and COVID-19 treatment.

#### Plant Metabolites Cross-Talking With Multiple Cellular Pathways

Flavonoids are a family of plant-originated polyphenolic compounds that regulate NF-κB to exert anti-inflammatory and anti-cancer effects (41), which might be promising to treat both COVID-19 and lung cancer.

Some flavonoids, such as epigallocatechin-3-Gallate, serve as NRF2 agonists to activate NRF2-HO-1 and NRF2/ARE pathways, thus reversing the COVID-19 progression (41). An *in silico* model has identified 14 flavonoids that could potentially bind to 3CLpro, an active catalytic site on SARS-CoV-2 (122), which may serve as a potential target for antiviral therapy. Moreover, previous studies confirmed the vital role of Hyperoside, one major flavonoid glycoside of *Zanthoxylum bungeanum*, in the induction of cell apoptosis and the inhibition of cell proliferation and migration in NSCLC (123). In breast cancer cells, Hyperoside enables NF-κB pathway deactivation *via* reducing intracellular ROS levels, which leads to downregulation of anti-apoptotic genes (*XIAP, Bcl-2*) and Bax accumulation, thereby promoting tumor cell apoptosis (124). Therefore, flavonoids, as potent anti-cancer and antiviral agents regulating ROS-mediated NF-κB signaling, deserve future research in COVID-19 and lung cancer treatment.

## Conclusion and Future Perspectives

In past years, studies have highlighted the importance of ROS in COVID-19 and lung cancer progression and different metabolic mechanisms modulating the production and scavenging of ROS, which assists in the in-depth knowledge of disease pathophysiology. Under oxidative stress, cells would regulate transcription factors to control various downstream antioxidant responses mainly *via* ROS-sensitive pathways. Here, we focus on the several pathways involved in ROS-induced pathogenesis, including redox-sensitive transcription factor NRF2, the hypoxia-induced factors HIF-1α, and the NF-κB signaling. Although some similarities are shared, the activity of these signaling molecules and their related crosstalk with ROS differ. The changes of NRF2-related pathways present opposite trends that NRF2 is inactivated in COVID-19 but activated in lung cancer. Specifically, the activation of NRF2 in lung cancer facilitates the immune escape of tumor cells; the downregulation of NRF2 in COVID-19 patients with lung cancer causes immunosuppressive effects to deteriorate the COVID-19 symptoms in lung cancer patients. HIF-1α upregulation in response to hypoxic stress in COVID-19 contributes to pathogenic effects in lung cancer, such as angiogenesis, metastasis, and cytokine storms. Moreover, hypoxia-induced HIF-1 might enhance PD-1 expression and lead to immune system escape based on the PD-1 pathway in both two diseases. COVID-19 might also lead to lung cancer *via* activating the inflammatory NF-κB pathway, considering its role in activating DCC. The lung cancer patients with COVID-19 probably present more severe symptoms compared to non-cancer patients, as the overactivated NF-κB pathway is critically associated with COVID-19 severity. However, the correlation between COVID-19 infection and pulmonary metastatic recurrence remains to be clarified.

More detailed understandings of these molecular mechanisms have allowed identifying novel therapeutic targets and the advancement of therapies that alter ROS levels based on these pathways. Many natural products, especially phytochemicals, are potent agents possessing both anti-inflammatory and anticancer effects *via* ROS-based cell killing, including flavonoids involved in several signaling pathways, and analogs/derivatives of these plants’ metabolites have been synthesized with lower toxicity and shown beneficial outcomes in preclinical use targeting lung cancer cells. Secondly, HIF-1α, the key regulator of hypoxia, induces the transcription expression of hundreds of hypoxia-responsive genes at low oxygen levels. Since hypoxia is important for inflammation and tumor growth, suppressing hypoxia-induced signaling pathways and changing oxygen exposure, such as hyperoxic treatment, provide attractive approaches to inhibit lung cancer metastasis and COVID-19 progression. Thirdly, recent studies suggested miRNAs were crucial in both lung cancer and COVID-19, thus receiving extensive attention in clinical usage.

Though we have recognized how ROS influences several molecular pathways in lung cancer, mechanisms and effects of potential and promising drugs targeting ROS-related signaling need to be further investigated before clinical applications. In contrast, as most attention is currently paid to study the immunological mechanisms and developing vaccines in COVID-19, effects of ROS on the pathogenesis and treatment of COVID-19 remain elusive. Future research aimed at investigating ROS-related pathways will probably decipher novel therapeutic targets and provide novel insights into specific drug design to treat and manage these devastating diseases.

## Author Contributions

JL designed the study and revised the manuscript. ZZhu and ZZheng wrote the manuscript. All authors contributed to the article and approved the submitted version.

## Funding

This work was supported by the Starting Fund of Zhejiang University to JL (grant number 130000-171207704/041).

## Conflict of Interest

The authors declare that the research was conducted in the absence of any commercial or financial relationships that could be construed as a potential conflict of interest.
